# Retrospective exploratory dual-center analysis of temporary transvenous cardiac pacing in cardiogenic shock

**DOI:** 10.1038/s41598-025-10364-9

**Published:** 2025-07-19

**Authors:** Clemens Walter von Musil, Valentina Riederer, Leonhard Pilsbacher, Carina Maria Paulus, Severin Rudinger, Sophia Bodlee, Jonas Gmeiner, Julius Fischer, Julius Steffen, Sven Peterß, Stefan Kääb, Moritz Sinner, Korbinian Lackermair, Martin Orban, Steffen Massberg, Clemens Scherer

**Affiliations:** 1 Department of Medicine I, LMU University Hospital, Ludwig-Maximilians-University (LMU) Munich, Marchioninistraße 15, 81377 Munich, Germany; 2 DZHK (German Centre for Cardiovascular Research), Munich Heart Alliance, Munich, Germany; 3 Department of Cardiac Surgery, LMU University Hospital, LMU Munich, Munich, Germany

**Keywords:** Cardiogenic shock, Acute myocardial infarction, Temporary cardiac pacing, Intensive care, Arrhythmia, Cardiac device therapy, Arrhythmias

## Abstract

Temporary transvenous cardiac pacing (TTP) is commonly used to manage hemodynamically compromising, drug-refractory brady- and tachyarrhythmias in the intensive care setting. Despite previous studies analyzing TTP treatment, data on its use in patients with cardiogenic shock (CS) remain limited. This retrospective exploratory analysis aimed to investigate the utilization of TTP in patients experiencing CS, with a particular focus on treatment characteristics, complication rates, predictive factors, and outcomes across different subgroups. We retrospectively included registry data from 184 patients who underwent TTP therapy from 1561 cases of CS treated at the Intensive Care Units (ICUs) of two university hospitals in Germany between 2010 and 2023. Bradycardia due to acute myocardial infarction was the primary indication for TTP implantation in patients with CS. The median duration of TTP therapy was 65 h, during which complications occurred in 12.0% of cases, 3.3% were classified as severe. We found that culprit lesions in the Right Coronary Artery (RCA) were more likely to necessitate TTP treatment in patients with acute myocardial infarction complicated by cardiogenic shock (AMI-CS) (OR 2.52, 95% CI 1.54–4.11, p < 0.001). In non-AMI-CS patients, age (OR 1.03, 95% CI 1.01–1.05, p < 0.005) and myocarditis (OR 3.21, 95% CI 1.19–8.64, p = 0.02) were associated with a higher incidence of TTP therapy during ICU treatment. Further studies are needed to validate these observations.

*Trial registration:* LMUshock registry (WHO International Clinical Trials Registry Platform Number DRKS00015860).

## Introduction

Cardiogenic shock (CS) is a life-threatening state of end-organ hypoperfusion due to a primary cardiac disorder necessitating pharmacological or mechanical circulatory support^1^. It is defined by both clinical findings and laboratory parameters, and is associated with a 30-day mortality rate of up to 50%^2–4^. CS is primarily caused by acute myocardial infarction (AMI); other etiologies include chronic heart failure, arrhythmias, myocarditis, and valvular causes^5^.

Temporary transvenous pacemaker (TTP) devices are leads that are mainly implanted in emergency settings in patients with life-threatening brady- or tachyarrhythmias. According to current guidelines, TTP is indicated for cases of reversible, hemodynamically compromising bradyarrhythmia refractory to pharmacological treatment^6^. TTP can serve as a bridge to the resolution of arrhythmia or permanent cardiac pacing when immediate implantation is not indicated or available^6,7^. TTP therapy can also be used in the management of refractory supraventricular and ventricular tachyarrhythmias^8^. A scoping review analyzing 32 studies found an overall mean in-hospital mortality rate of 14.5% in patients with TTP therapy of all causes, indicating a patient population with high morbidity^9^. Furthermore, it was previously shown that in-hospital mortality in patients with AMI requiring TTP was 6.5-fold higher than that of other patients with bradyarrhythmia. The authors hypothesized that the severity of myocardial infarction and the consequent development of conduction disturbances may be directly related to increased mortality^7^.

Despite previous studies analyzing TTP treatment, data on its use in patients with cardiogenic shock (CS) remain limited. This study aims to investigate the utilization of TTP in CS patients, examining treatment characteristics, complication rates, predictive factors, and outcomes across different subgroups.

## Methods

### LMUshock registry and patient population

The LMUshock registry includes patients treated for cardiogenic shock (SCAI shock classification C-E^10^) at two university hospitals of Ludwig-Maximilians-University (LMU), Munich, Germany, since January 2010. It is listed on the WHO International Clinical Trials Registry Platform (DRKS00015860). Data collection complies with the principles outlined in the Declaration of Helsinki and with German data protection law. Informed consent was obtained from all patients or their legal representatives when possible. In cases where patients were unable to provide consent and died before consent could be obtained, data were included in an anonymized form in accordance with the approval granted by the Ethics Committee of the Medical Faculty of LMU Munich (IRB number: 18–001).

Cardiogenic shock was defined following ESC guidelines, the IABP shock II trial and CULPRIT-SHOCK trial as a state of end organ hypoperfusion due to a primary cardiac cause with systolic blood pressure < 90 mmHg persisting for ≥ 30 min or the requirement of pharmacological and/or mechanical cardiac support to sustain a systolic blood pressure above 90 mmHg. Additionally, clinical findings such as oliguria, altered mental status, or cold and clammy extremities, as well as laboratory parameters including elevated serum lactate levels above 2 mmol/l or increased renal retention, were considered^1,11,12^.

For our analysis, we examined data from the LMUshock registry, including all patients over 18 years who received temporary transvenous pacemaker (TTP) treatment during their ICU stay. Patients with epicardial temporary pacemakers were excluded from this analysis.

### Temporary transvenous cardiac pacing (TTP)

In our centers, temporary transvenous pacing (TTP) was performed using different approaches depending on the clinical setting. In the ICU setting, Arrow® Balloon Temporary Pacing Catheters (Teleflex™, Morrisville, NC, USA) were inserted at bedside via the jugular vein under ultrasound guidance. A Tuohy locking sheath was used to secure the catheter in place. In the catheterization lab, Supreme™ Quadripolar Electrophysiology Catheters (Abbott Cardiovascular, Plymouth, MN, USA) were implanted via the femoral vein under fluoroscopic guidance. If TTP was required for a more extended period, catheters initially placed through the femoral vein were subsequently replaced via jugular vein access.

All procedures were performed by experienced cardiologists, anesthesiologists, or intensive care specialists in the emergency department (ED) or on the intensive care unit (ICU). For ultrasound-guided bedside implantations, lead placement was verified using chest X-ray. One center is led by cardiologists with 24/7 on-site cardiology coverage, while the other is managed by anesthesiologists and internists. In both centers, consultation with an electrophysiologist and an intensive care attending physician is available around the clock. TTPs were implanted in the catheterization lab by interventional cardiologists if another procedure was already being conducted there or if implantation was only feasible in that location due to technical difficulties.

### Statistical analysis

R software (version 4.3.2, The R Foundation for Statistical Computing, Vienna, Austria, 2023) and the following R packages were used for all statistical computations: “tableone” (version 0.13.2, Yoshida K., Bartel A., 2022), “survival” (version 3.5–8, Therneau T., 2024), “epitools” (version 0.5–10.1, Aragon T., 2020), “dplyr” (version 1.1.4, Wickham H., François R., Henry L., Müller K., Vaughan D., 2023) “survminer” (version 0.4.9, Kassambara A., Kosinski M., Biecek P., 2021), “ggfortify” (version 0.4.17, Yuan T., Masaaki H., Wenxuan L., 2016), “ggplot2” (version 3.5.1, Wickham H., Springer, New York, 2016), “MASS” (version 7.3–61, Venables, W. N. & Ripley, B. D., Springer, New York, 2002) and “mice” (version 3.16.0, van Buuren S., Groothuis-Oudshoorn K., 2011).

Normally distributed variables were presented as mean with standard deviation, while non-normally distributed variables were reported as median with interquartile ranges. The Shapiro-Wilks test was used to assess whether variables were distributed normally. Categorical variables were depicted as absolute numbers with percentages and Chi-squared or Fisher’s exact test were used for comparison. The odds ratio test was used for subsequent analysis of categorical variables. The log-rank test was used to compare the survival distribution of groups. Kaplan–Meier curves were used to visualize the survival over time. Missing data for laboratory parameters was imputed by the R package “mice”. Univariable and multivariable regression analyses were performed to identify independent predictors of TTP therapy. Variables for multivariable analysis were selected with the “stepAIC” function (“MASS” package) with backwards direction. Universal significance was set at p < 0.05.

## Results

### Patient characteristics

At the time of our analysis, data from 1561 LMUshock registry patients were available. Of these, 184 (11.8%) patients underwent treatment with TTP between January 2010 and November 2023. The majority of patients treated with TTP, 129 (70.1%), were male, with a mean age of 69.0 years (SD 13.4 years). AMI emerged as the most prevalent etiology of CS in TTP patients, accounting for 59.2% of the cases, of which 48.6% presented with ST-elevation myocardial infarction (STEMI) and 51.4% with non-ST-elevation myocardial infarction (NSTEMI). This was followed by primary arrhythmias (11.4%) and valvular causes (11.4%). 69.6% of the patients treated with TTP experienced cardiac arrest (CA), and in 25.5% of the patients, out-of-hospital cardiac arrest (OHCA) occurred. Venous-arterial extracorporeal life support (VA-ECLS) was necessary in 31.0% of the cases, left ventricular coaxial pumps were implanted in 13.0%. Tables 1 and 2 present additional key patient information.

**Table 1:** LMUshock registry: patient characteristics.

	Non-TTP (n = 1377)	TTP (n = 184)	p value
Sex, n (%)			0.40
Female	367 (26.7)	55 (29.9)	
Male	1010 (73.3)	129 (70.1)	
Age, mean (SD)	65.09 (15.58)	68.98 (13.44)	< 0.01*
Cardiovascular risk factors, n (%)			
Hypertension	877 (63.7)	137 (74.5)	< 0.01*
Diabetes	387 (28.1)	56 (30.4)	0.01*
Hyperlipidemia	594 (43.1)	74 (40.2)	0.07
Smoking			0.10
Active smoker	289 (21.0)	43 (23.4)	
ex-smoker	293 (21.3)	26 (14.1)	
Positive family history	231 (16.8)	18 (9.8)	0.03*
History of PCI or CABG, n (%)	395 (28.7)	44 (23.9)	0.21
Chronic dialysis before admission, n (%)	48 (3.5)	9 (4.9)	0.16
BMI, median [IQR]	26.23 [23.63, 29.39]	27.10 [24.17, 30.52]	0.11
SCAI at admission, n (%)			0.77
A	11 (0.8)	2 (1.1)	
B	53 (3.8)	5 (2.7)	
C	395 (28.7)	49 (26.6)	
D	256 (18.6)	30 (16.3)	
E	662 (48.1)	98 (53.3)	

All values are presented as mean/median and confidence intervals, or absolute values and percentages.

Positive family history: cardiovascular event in first-degree relatives before the age of 55 (males) or 65 (females), PCI = percutaneous coronary intervention, CABG = coronary artery bypass graft, SCAI Classification = Society for Cardiovascular Angiography & Interventions Stages of Cardiogenic Shock,

* = p-values < 0.05 were considered statistically significant.

**Table 2:** Etiology of cardiogenic shock in LMUshock registry.

Etiology of CS, n (%)	Non-TTP therapy (n = 1377)	TTP therapy (n = 184)	p < 0.01*
Myocardial infarction	663 (48.1)	109 (59.2)	
Cardiomyopathy	242 (17.6)	8 (4.3)	
Arrhythmia	138 (10.0)	21 (11.4)	
Intoxication	7 (0.5)	2 (1.1)	
Myocarditis	25 (1.8)	8 (4.3)	
Other	172 (12.5)	13 (7.1)	
Unknown	7 (0.5)	2 (1.1)	
Valvular	123 (8.9)	21 (11.4)	

All values are presented as absolute values and percentages.

* = p-values < 0.05 were considered statistically significant.

772 of 1561 patients (49.5%) were admitted to the ICU with acute myocardial infarction complicated by cardiogenic shock (AMI-CS). 10.8% of these patients were treated with TTP because of bradycardia secondary to myocardial infarction (AMI-CS-TTP). The mean age of this group was 68.2 years (SD 10.4). 51.8% of the patients presented with ST-segment elevation myocardial infarction (STEMI), while 48.2% suffered non-ST-segment elevation myocardial infarction (NSTEMI). 26 patients were admitted to the ICU due to AMI-CS but received TTP therapy due to a reason not directly related to AMI (AMI-CS-non-TTP). Additional patient information is outlined in Table 3.

**Table 3:** Characteristics of AMI-CS patients.

	AMI-CS-non-TTP (n = 689)	AMI-CS-TTP (n = 83)	p-value
Sex, n (%)			1.00
Female	167 (24.2)	20 (24.1)	
Male	522 (75.8)	63 (75.9)	
Age, mean (SD)	67.36 (12.85)	68.16 (10.42)	0.59
Etiology cardiogenic shock, n (%)			0.36
NSTEMI	291 (42.2)	40 (48.2)	
STEMI	398 (57.8)	43 (51.8)	
Cardiovascular risk factors, n (%)			
Hypertension	475 (68.9)	65 (78.3)	0.20
Diabetes	215 (31.2)	29 (34.9)	0.66
Hyperlipidemia	319 (46.3)	32 (38.6)	0.32
Smoking			0.53
Active smoker	190 (27.6)	26 (31.3)	
Ex-smoker	119 (17.3)	10 (12.0)	
Positive family history	111 (16.1)	5 (6.0)	0.05
History of PCI or CABG, n (%)	200 (29.0)	23 (27.7)	0.90
Chronic dialysis before admission, n (%)	19 (2.8)	3 (3.6)	0.37
BMI, median [IQR]	26.64 [24.28, 29.39]	27.50 [23.70, 30.86]	0.60
SCAI at admission, n (%)			0.60
A	7 (1.0)	1 (1.2)	
B	28 (4.1)	1 (1.2)	
C	160 (23.2)	20 (24.1)	
D	118 (17.1)	11 (13.3)	
E	376 (54.6)	50 (60.2)	
Laboratory parameters at admission, median [IQR]			
Arterial pH	7.27 [7.17, 7.35]	7.24 [7.16, 7.36]	0.57
Lactate [mmol/l]	6.40 [2.70, 11.70]	7.25 [3.82, 12.40]	0.17
Hemoglobin [mg/dl]	10.90 [10.10, 12.80]	10.70 [10.10, 12.30]	0.26
Troponin T (hs) [ng/ml]	9.11 [2.73, 51.55]	7.97 [2.78, 19.60]	0.20
Creatine Kinase [U/l]	1281.50 [448.00, 3284.25]	2119.00 [1315.75, 3329.75]	0.10
Creatinine, Jaffé [mg/dl]	1.40 [1.10, 1.80]	1.30 [1.10, 1.80]	0.96
Culprit lesions causing AMI-CS, n (%)			< 0.01*
Left anterior descending artery (LAD)	326 (47.3)	32 (38.6)	
Left circumflex artery (LCx)	100 (14.5)	12 (14.5)	
Left main artery (LM)	94 (13.6)	5 (6.0)	
Right coronary artery (RCA)	133 (19.3)	31 (37.3)	
Unknown/NA	36 (5.3)	3 (3.6)	

All values are presented as mean/median and confidence intervals, or absolute values and percentages.

NSTEMI = non-ST-segment-elevation myocardial infarction, STEMI = ST-elevation myocardial infarction, Positive family history: cardiovascular event in first-degree relatives before the age of 55 (males) or 65 (females), PCI = percutaneous coronary intervention, CABG = coronary artery bypass graft,

SCAI Classification = Society for Cardiovascular Angiography & Interventions Stages of Cardiogenic Shock, pH = potential of hydrogen.

* = p-values < 0.05 were considered statistically significant.

Of the 1561 patients, 789 (50.5%) were treated with non-AMI-CS. 9.5% of all non-AMI-CS patients needed TTP therapy during their ICU stay. The mean age of this group was 70.5 years (SD 16.8). The most common etiologies causing non-AMI-CS were cardiomyopathies (31.7%), followed by primary arrhythmias (20.2%) and valvular pathologies (18.3%). Additional patient information is detailed in Table 4.

**Table 4:** Characteristics of non-AMI-CS patients.

	Non-AMI-CS-non-TTP (n = 714)	Non-AMI-CS-TTP (n = 75)	p-value
Sex, n (%)			0.06
Female	205 (28.7)	30 (40.0)	
Male	509 (71.3)	45 (60.0)	
Age, mean (SD)	62.98 (17.45)	70.52 (16.78)	< 0.01*
Etiology of cardiogenic shock, n (%)			< 0.01*
Cardiomyopathy	242 (33.9)	8 (10.7)	
Arrhythmia	138 (19.3)	21 (28.0)	
Intoxication	7 (1.0)	2 (2.7)	
Myocarditis	25 (3.5)	8 (10.7)	
Valvular	123 (17.2)	21 (28.0)	
Other	172 (24.1)	13 (17.3)	
Unknown	7 (1.0)	2 (2.7)	
Cardiovascular risk factors, n (%)			
Hypertension	423 (59.2)	51 (68.0)	0.05
Diabetes	177 (24.8)	22 (29.3)	< 0.01*
Hyperlipidemia	289 (40.5)	28 (37.3)	0.30
Smoking			0.02*
Active smoker	108 (15.1)	8 (10.7)	
Ex-smoker	179 (25.1)	11 (14.7)	
Positive family history	124 (17.4)	9 (12.0)	0.33
History of PCI or CABG, n (%)	202 (28.3)	14 (18.7)	0.10
Chronic dialysis before admission, n (%)	29 (4.1)	6 (8.0)	0.23
BMI, median [IQR]	25.95 [23.15, 29.39]	27.18 [24.20, 29.75]	0.20
SCAI at admission, n (%)			0.89
A	4 (0.6)	1 (1.3)	
B	26 (3.6)	3 (4.0)	
C	239 (33.5)	23 (30.7)	
D	144 (20.2)	13 (17.3)	
E	299 (41.9)	35 (46.7)	
Laboratory parameters at admission, median [IQR]			
Arterial pH	7.34 [7.23, 7.41]	7.33 [7.23, 7.40]	0.57
Lactate [mmol/l]	3.30 [2.00, 7.40]	3.20 [2.22, 6.60]	0.88
Hemoglobin [mg/dl]	10.90 [10.00, 12.60]	10.30 [10.00, 11.43]	0.03*
Troponin T (hs) [ng/ml]	81.00 [18.60, 234.00]	86.00 [9.32, 246.50]	0.82
Creatine Kinase [U/l]	136.50 [65.00, 396.50]	169.00 [113.00, 251.50]	0.50
Creatinine, Jaffé [mg/dl]	1.50 [1.10, 2.30]	1.50 [1.20, 2.20]	0.86

All values are presented as mean/median and confidence intervals, or absolute values and percentages.

Positive family history: cardiovascular event in first-degree relatives before the age of 55 (males) or 65 (females), PCI = percutaneous coronary intervention, CABG = coronary artery bypass graft,

SCAI Classification = Society for Cardiovascular Angiography & Interventions Stages of Cardiogenic Shock, pH = potential of hydrogen.

* = p-values < 0.05 were considered statistically significant.

### Characteristics of TTP therapy in cardiogenic shock

Bradycardia secondary to acute myocardial ischemia represented the primary indication for pacemaker implantation, with 45.1% of the cases. Conduction disturbances within interventional cardiac treatment accounted for 16.3%, of which Transcatheter Aortic Valve Replacement (TAVR) represented 90.0% and Transcoronary Ablation of Septal Hypertrophy (TASH) accounted for 6.7% of the cases. In 3.3%, bradycardia due to an irritation of Tawara branches during an implantable cardioverter-defibrillator (ICD) explantation necessitated TTP therapy. Attempts to terminate or prevent tachyarrhythmia by overdrive pacing, which involves avoiding R-to-T phenomena, represented 8.7% of all cases. In 4.9% of the patients, TTP was implanted because of bradycardia due to obstructive reasons, such as pulmonary artery embolism, followed by arrhythmia within myocarditis (4.9%) and sinus node dysfunction (3.8%). Further information is outlined in Table 5.

**Table 5:** Reason for temporary transvenous pacemaker (TTP) implantation in CS.

Reason for TTP implantation, n (%)	TTP patients (n = 184)
Myocardial ischemia	83 (45.1)
AV block III° due to MI	32 (17.4)
Sinus bradycardia due to MI	17 (9.2)
Asystole/unknown block due to MI	16 (8.7)
Others/unknown	18 (9.8)
Conduction disturbance within interventional cardiac treatment	30 (16.3)
AV block III°	20 (10.9)
Sinus bradycardia	1 (0.5)
Asystole/unknown block	1 (0.5)
Others/unknown	8 (4.4)
Overdrive pacing	16 (8.7)
Prevention of R-on-T phenomena	2 (1.1)
Treatment of tachyarrhythmias	14 (7.6)
Bradycardia due to adverse drug reaction	10 (5.4)
Bradycardia due to obstructive reasons	9 (4.9)
Arrhythmia within myocarditis	9 (4.9)
Bradycardia due to hypothermia	7 (3.8)
Sinus node dysfunction	7 (3.8)
Hyperkalemia	4 (2.2)
Other/unknown	9 (4.9)

Third-degree atrioventricular block (AV block III) was the most prevalent ECG rhythm observed before implantation, documented in 38.0% of the cases, followed by sinus bradycardia (18.5%), asystole/unknown block (14.7%), and junctional rhythm (7.1%). Further details are outlined in Table 6.

**Table 6:** ECG rhythms before TTP implantation.

ECG rhythm	All TTP patients (n = 184)	Ischemia (n = 83)	Conduction disturbance within int. card. treatment (n = 30)	Over-drive pacing (n = 16)	Adverse drug reaction (n = 10)	Bradycardia due to obstructive reasons (n = 9)
AV block III°	70 (38.0)	32 (38.6)	20 (66.7)	0 (0.0)	3 (30.0)	3 (33.3)
Sinus bradycardia	34 (18.5)	17 (20.5)	1 (3.3)	2 (12.5)	3 (30.0)	2 (22.2)
Asystole/unknown block	27 (14.7)	16 (19.3)	1 (3.3)	0 (0.0)	2 (20.0)	2 (22.2)
Junctional rhythm	13 (7.1)	9 (10.8)	0 (0.0)	0 (0.0)	0 (0.0)	0 (0.0)
Atrial fibrillation	8 (4.3)	3 (3.6)	2 (6.7)	0 (0.0)	2 (20.0)	1 (11.1)
Vent. fib	7 (3.8)	0 (0.0)	0 (0.0)	7 (43.8)	0 (0.0)	0 (0.0)
AV block II°	6 (3.3)	1 (1.2)	3 (10.0)	0 (0.0)	0 (0.0)	1 (11.1)
Atrial flutter	2 (1.1)	0 (0.0)	0 (0.0)	1 (6.2)	0 (0.0)	0 (0.0)
VT with pulse	3 (1.6)	0 (0.0)	0 (0.0)	3 (18.8)	0 (0.0)	0 (0.0)
VT without pulse	3 (1.6)	0 (0.0)	0 (0.0)	3 (18.8)	0 (0.0)	0 (0.0)
Sinoatrial block	5 (2.7)	2 (2.4)	0 (0.0)	0 (0.0)	0 (0.0)	0 (0.0)
Sinus rhythm	1 (0.5)	1 (1.2)	0 (0.0)	0 (0.0)	0 (0.0)	0 (0.0)
Other	1 (0.5)	1 (1.2)	0 (0.0)	0 (0.0)	0 (0.0)	0 (0.0)
Unknown	4 (2.2)	1 (1.2)	3 (10.0)	0 (0.0)	0 (0.0)	0 (0.0)

	Arrhythmia within myo- carditis (n = 9)	Bradycardia due to hypo-thermia (n = 7)	Sinus node dysfunction (n = 7)	Hyper-kalemia (n = 4)	other/ unknown (n = 9)	
AV block III°	6 (66.7)	0 (0.0)	0 (0.0)	2 (50.0)	4 (44.4)	
Sinus bradycardia	2 (22.2)	6 (85.7)	1 (14.3)	0 (0.0)	0 (0.0)	
Asystole/unknown block	1 (11.1)	0 (0.0)	2 (28.6)	0 (0.0)	3 (33.3)	
Junctional rhythm	0 (0.0)	0 (0.0)	3 (42.9)	1 (25.0)	0 (0.0)	
Atrial fibrillation	0 (0.0)	0 (0.0)	0 (0.0)	0 (0.0)	0 (0.0)	
Vent. fib	0 (0.0)	0 (0.0)	0 (0.0)	0 (0.0)	0 (0.0)	
AV block II°	0 (0.0)	0 (0.0)	0 (0.0)	0 (0.0)	1 (11.1)	
Atrial flutter	0 (0.0)	1 (14.3)	0 (0.0)	0 (0.0)	0 (0.0)	
VT with pulse	0 (0.0)	0 (0.0)	0 (0.0)	0 (0.0)	0 (0.0)	
VT without pulse	0 (0.0)	0 (0.0)	0 (0.0)	0 (0.0)	0 (0.0)	
Sinoatrial block	0 (0.0)	0 (0.0)	1 (14.3)	1 (25.0)	1 (11.1)	
Sinus rhythm	0 (0.0)	0 (0.0)	0 (0.0)	0 (0.0)	0 (0.0)	
Other	0 (0.0)	0 (0.0)	0 (0.0)	0 (0.0)	0 (0.0)	
Unknown	0 (0.0)	0 (0.0)	0 (0.0)	0 (0.0)	0 (0.0)	

All values are presented as absolute values and percentages, int. card. treatment = interventional cardiac treatment, AV block = atrioventricular block, Vent. fib. = ventricular fibrillation, VT = ventricular tachycardia.

**Fig. 1:**
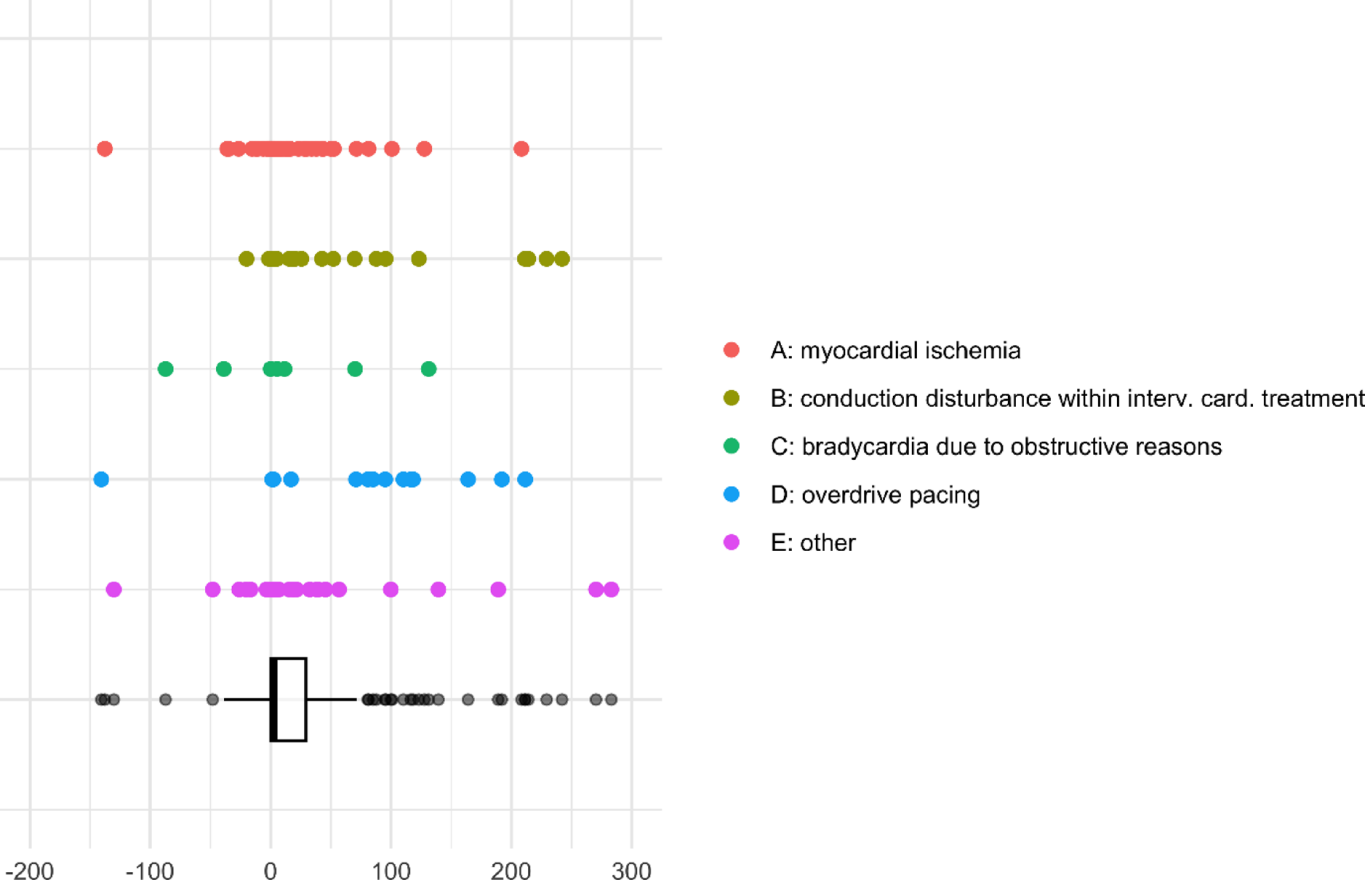
Time of TTP implantation regarding ICU admission. Time of TTP implantation distribution (hours) regarding ICU admission for various TTP indications. Myocardial ischemia (red): median 1.3 h [− 1 h, 13.1 h], conduction disturbance within interventional cardiac treatment (yellow): median 16.9 h [0.3 h, 65.3 h], bradycardia due to obstructive reasons (green): median 0.0 h [− 38.9 h, 11.5 h], overdrive pacing (blue): median 82.9 h [2.0 h, 116.8 h], other (violet): median 4.2 h [0.1 h, 39.4 h], overall (black): median 3.1 h [0.0 h, 35.0 h].

The overall median time of TTP implantation was 3.1 [0.0, 35.0] hours after ICU admission. In rare cases, patients received primary TTP implantation in external hospitals before being admitted to our ICUs. Figure 1 presents the distribution of implantation time for different TTP indications.

### Complications in TTP therapy in cardiogenic shock

The median duration of TTP therapy was 65 h (IQR 20.0–114.3 h), during which 23 complications occurred in 22 patients (12.0%). The most common complication was dislocation of the intraventricular pacemaker lead/loss of capture (10 cases), followed by arrhythmias during implantation (3 cases), and infections (3 cases). All complications are detailed in Table 7. Notably, in 6 cases (3.3%), cardiopulmonary resuscitation (CPR) was required due to a TTP device-related complication. No patient died of a complication directly attributed to TTP treatment.

**Table 7:** Complications within TTP treatment.

Complications, n (%)	All complications within TTP therapy (n = 23)	Severe complications necessitating CPR within TTP treatment (n = 6)
Implantation		
Cardiac perforation	1 (0.5)	0 (0.0)
Tension pneumothorax	1 (0.5)	0 (0.0)
Mispuncture of access vessel	1 (0.5)	0 (0.0)
Arrhythmia	3 (1.6)	0 (0.0)
Failed implantation attempt	2 (1.1)	0 (0.0)
During therapy		
Dislocation/loss of capture	10 (5.4)	5 (2.7)
Infection	3 (1.6)	0 (0.0)
Arrhythmia	2 (1.1)	1 (0.5)

All values are presented as absolute values and percentages.

### Predictors of TTP therapy in AMI-CS and non-AMI-CS

Univariable and multivariable correlation analysis revealed no significant association between age, sex, or laboratory parameters such as serum lactate, arterial pH, serum creatine kinase, or creatinine at admission and the necessity for TTP treatment in AMI-CS patients. However, a statistically significant correlation was observed between the culprit lesion causing AMI-CS and the necessity for TTP implantation (p < 0.005). Subsequent analysis revealed no significant associations between culprit lesions in the Left Anterior Descending Artery (OR 0.67, 95% CI 0.41–1.08, p = 0.1), Left Circumflex Artery (OR 0.93, 95% CI 0.46–1.72, p = 0.82) or the Left Main coronary artery (OR 0.40, 95% CI 0.12–1.01, p = 0.06) and the necessity of TTP treatment in CS. However, a significant association was identified between culprit lesions in the Right Coronary Artery (RCA) and the need for TTP treatment (OR 2.47, 95% CI 1.50–4.02, p < 0.001). In multivariable regression analysis, culprit lesions in the RCA were identified as a strong independent predictor of TTP therapy in AMI-CS patients (OR 2.52, 95% CI 1.54–4.11, p < 0.001).

In non-AMI-CS patients, age (OR 1.03, 95% CI 1.01–1.05, p < 0.005) and specific etiologies of cardiogenic shock were identified as independent predictors of TTP therapy. Myocarditis was associated with a higher incidence (OR 3.21, 95% CI 1.19–8.64, p = 0.02), while cardiomyopathies were associated with a lower incidence (OR 0.26, CI 0.11–0.63, p < 0.005) of TTP therapy during intensive care, as compared to the “arrhythmia” reference category in multivariable regression analysis.

### Outcome of TTP therapy in cardiogenic shock

**Fig. 2:**
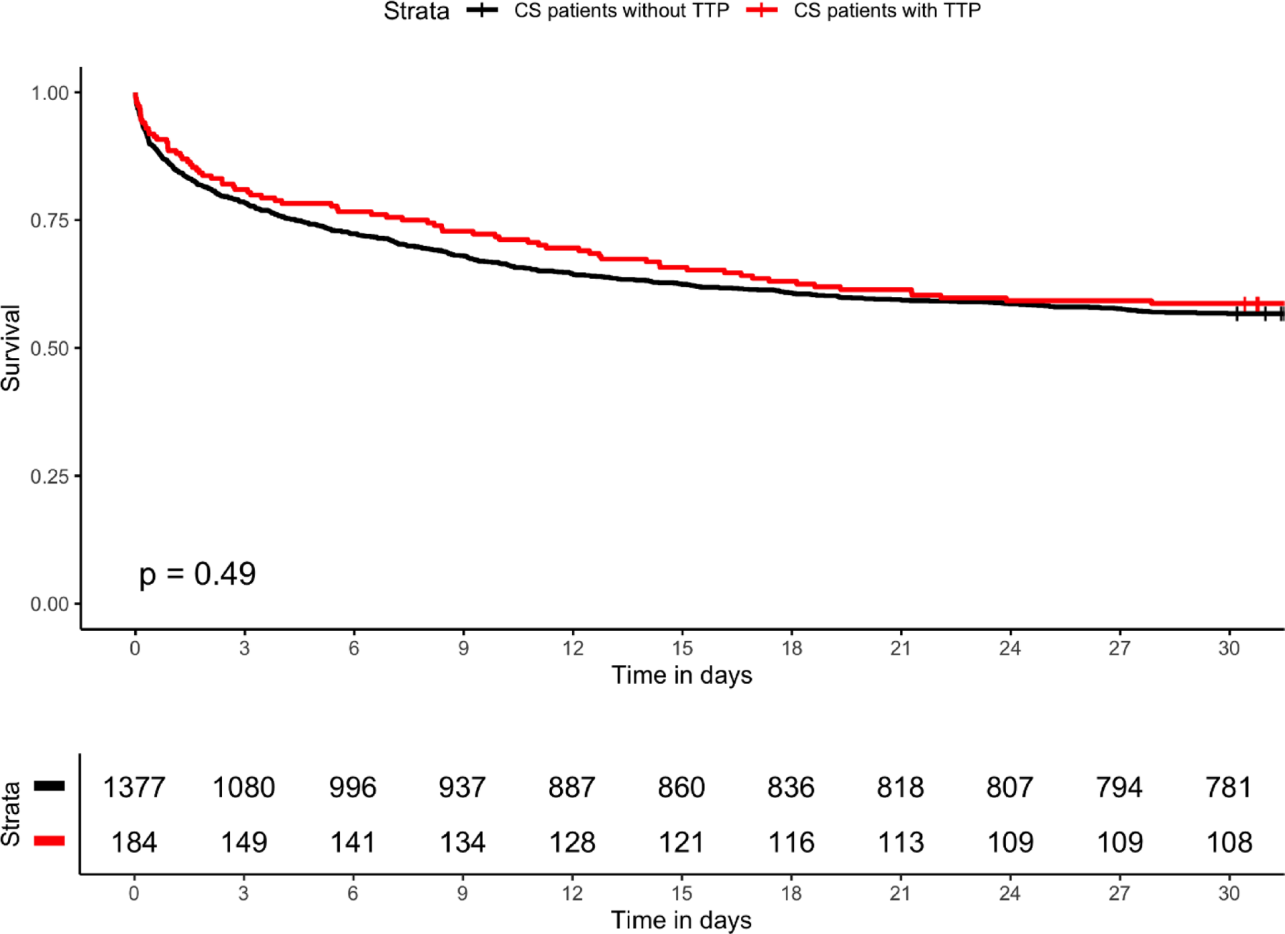
30-day survival in CS patients with and without TTP treatment. Cumulative survival curves for patients with cardiogenic shock of all causes with (red) and without (black) TTP treatment for 30 days from ICU admission. p-value = 0.49.

The 30-day survival rate was 58.7% among all patients who underwent TTP treatment. There was no significant difference in 30-day survival between TTP patients and non-TTP patients in cardiogenic shock (56.7% vs. 58.7%, p = 0.49, Fig. 2). However, a significant difference was seen in the 30-day survival between patients treated with TTP for bradycardia secondary to AMI-CS (AMI-CS-TTP) and non-AMI-CS-TTP patients (49.4% vs. 66.3%, p < 0.005, Fig. S1).

**Fig. 3:**
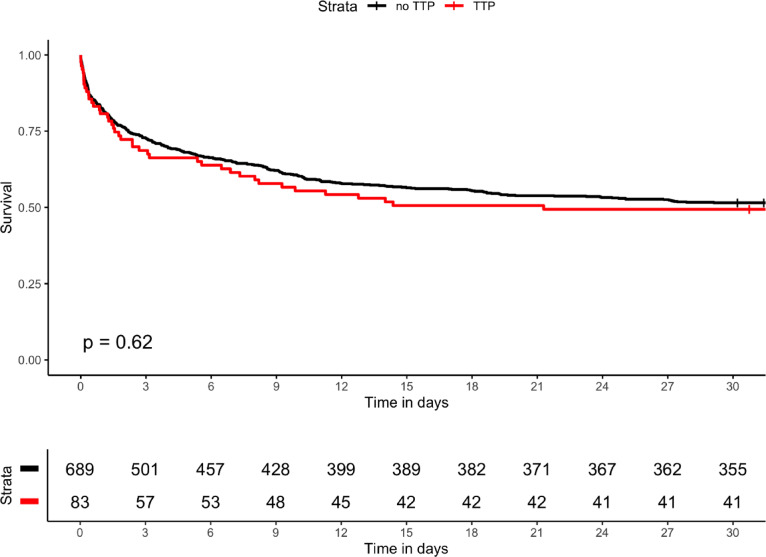
30-day survival in AMI-CS with and without TTP treatment. Cumulative survival curves for patients with acute myocardial infarction complicated by cardiogenic shock with (red) and without (black) TTP treatment for 30 days from ICU admission. p-value = 0.62.

In AMI-CS patients, we observed no significant difference in 30-day survival between AMI-CS-TTP and AMI-CS-non-TTP patients (49.4% vs. 51.5%, p = 0.62, Fig. 3).

Permanent pacemaker implantation was necessary in 20.7% of all TTP patients, and implantable cardioverter-defibrillator (ICD) devices were implanted in 7.6%. TTP device explantation due to the absence of bradycardia occurred in 40.2% of the cases, while 31.0% of the patients died before TTP removal was possible.

In AMI-CS-TTP patients, permanent pacemaker implantation was necessary in 10.8% of the cases (vs. 28.7% in non-AMI-CS-TTP patients), and ICD devices were implanted in 3.6% (vs. 10.9% in non-AMI-CS-TTP patients). TTP device explantation due to the absence of bradycardia occurred in 44.6% (vs. 36.6% in non-AMI-CS-TTP patients) of the cases. In comparison, 41.0% of the AMI-CS-TTP patients (vs. 22.8% in non-AMI-CS-TTP patients) died due to the progression of cardiogenic shock before TTP removal was possible. TTP explantation distribution differed significantly (p < 0.01) between AMI-CS-TTP and non-AMI-CS-TTP patients (Table 8). In AMI-CS patients surviving TTP treatment, permanent pacemaker or ICD implantation was necessary in 51.3% of non-AMI-CS-TTP cases, compared to 24.5% in AMI-CS-TTP cases (p < 0.005).

**Table 8:** TTP explantation in non-AMI-CS-TTP and AMI-CS-TTP patients.

Reason for TTP explantation	All TTP patients (n = 184)	non-AMI-CS-TTP (n = 101)	AMI-CS-TTP (n = 83)	p < 0.01*
Absence of bradycardia	74 (40.2)	37 (36.6)	37 (44.6)	
Death	57 (31.0)	23 (22.8)	34 (41.0)	
Permanent pacemaker implantation	38 (20.7)	29 (28.7)	9 (10.8)	
ICD implantation	14 (7.6)	11 (10.9)	3 (3.6)	
Unknown	1 (0.5)	1 (1.0)	0 (0.0)	

All values are presented as absolute values and percentages, ICD = implantable cardioverter-defibrillator.

* = p-values < 0.05 were considered statistically significant.

### Overdrive pacing using TTP in cardiogenic shock

In our sample, 16 patients (8.7%) were treated with TTP overdrive pacing to terminate or prevent tachyarrhythmias by avoiding R-on-T phenomena. The most frequently documented ECG rhythm before TTP implantation was ventricular fibrillation (VF), at 43.8%, followed by ventricular tachycardia (VT) at 37.5%. Further details are shown in Table 6. In 4 cases (25.0%), tachyarrhythmias were observed despite TTP overdrive pacing therapy; however, no other complications were documented in TTP overdrive pacing cases.

In 53.0% of the cases, the implantation of ICD or permanent pacemaker devices was necessary. Explantation due to absence of the arrhythmia was possible in 23.5%. Further, 23.5% died with TTP before removal was possible.

## Discussion

This study provides first insights into various aspects of temporary transvenous pacing (TTP) therapy in patients with cardiogenic shock (CS), with a specific focus on its utilization in cases of acute myocardial infarction complicated by CS (AMI-CS). While published studies show a diverse patient population receiving TTP for various indications, the role of TTP in CS has not been well characterized.

Our data show that bradycardia secondary to AMI is the most common reason for TTP implantation in CS, underlining the significance of myocardial infarction as a primary etiological factor in CS.

Our cohort showed an overall complication rate of 12%, mostly due to lead dislodgement or loss of capture. While these complications were relatively infrequent, they underscore the importance of vigilant monitoring and management of TTP therapy, particularly in patients with severe conditions such as cardiogenic shock. While the literature cites complication rates up to 94%, more recent data show a downward trend, indicating improved procedural safety^9^.

It is noteworthy that data on TTP complications exhibit considerable variability across studies due to diverse settings and factors, such as the medical specialty involved in implantation, the environment, the access vessel used, and the classification of complications, making direct comparisons challenging. To improve reliability and generalizability, further studies involving diverse patient populations are needed. Additionally, the implementation of standardized protocols for reporting complications would support more consistent and comprehensive comparisons across studies. To minimize complications related to TTP in patients with cardiogenic shock, several strategies should be considered. Ultrasound- or fluoroscopy-guided lead placement, combined with assessment of the lead position, can enhance procedural accuracy and reduce complications. Adherence to standard operating procedures (SOPs) for insertion, maintenance, and monitoring could improve consistency and safety. Involving experienced operators or specialized pacing teams could further reduce the risk of procedural errors. Regular device checks, including lead positioning and pacing thresholds, are crucial for detecting malfunctions early.

Our analysis revealed a significant association between culprit lesions in the Right Coronary Artery (RCA) and the necessity for TTP treatment in AMI-CS. This finding suggests that the location of acute myocardial infarction, together with its severity, may influence the development of potential life-threatening arrhythmias in CS. Our findings align with anatomical evidence suggesting that the cardiac conduction system is primarily supplied by the RCA and the observation that right ventricular AMI commonly leads to bradyarrhythmias^13,14^. To further explore the role of culprit lesions in the RCA in patients with AMI-CS, prospective studies with larger cohorts could help validate the link between RCA lesions and the necessity for TTP, as well as assess the impact of lesion localization and severity. Additionally, comparative studies on TTP protocols could help to optimize therapy strategies.

We found no significant difference in 30-day survival rates between patients who did and did not require TTP therapy in AMI-CS. However, we did see a significantly higher survival rate in non-AMI-CS patients undergoing TTP treatment compared to AMI-CS patients treated with TTP. This observation is consistent with the previously reported higher mortality rate in TTP patients suffering acute myocardial infarction^7^.

The reasons for device explantation differed significantly between subgroups. In AMI-CS patients, TTP often addressed transient arrhythmias not requiring long-term pacing, while non-AMI-CS patients more frequently progressed to permanent pacemaker or ICD implantation. This suggests differences in arrhythmia reversibility based on the underlying cause of CS.

In conclusion, TTP plays an important role in the management of drug-refractory, hemodynamically compromising arrhythmias in the ICU setting. Our data demonstrate that TTP therapy is an effective and safe treatment option, with a low incidence of severe complications. Further studies are needed to validate our findings.

### Limitations

This exploratory analysis has several limitations. Firstly, it is a retrospective, dual-center study, which may limit the reliability of our findings. Prospective trials with larger populations, conducted in diverse settings, are necessary to validate our findings. The patients included in our analysis were in the acute phase of cardiogenic shock, which may introduce bias and make the correlation to TTP treatment questionable. Additionally, some of our findings, such as specific complications, may be underestimated due to incomplete documentation.

## Supplementary Information

Supplementary Information.

## Data Availability

The dataset analyzed during this study is not publicly available due to ethical committee regulations but might be available from the corresponding author on reasonable request and provided that ethical committee approves data sharing.
