# Supplementary material for: Retrospective exploratory dual-center analysis of temporary transvenous cardiac pacing in cardiogenic shock
Source: Sci Rep. 2025 Jul 19;15:26241. doi: 10.1038/s41598-025-10364-9 (PMC12276278; doi:10.1038/s41598-025-10364-9)

**Supplementary material**

**Retrospective exploratory dual-center analysis of temporary transvenous cardiac pacing in cardiogenic shock**

Clemens Walter von Musil^1^ (#), Valentina Riederer^1^ (#), Leonhard Pilsbacher^1^, Carina Maria Paulus^1^,

Severin Rudinger^1^, Sophia Bodlee^1^, Jonas Gmeiner^1^, Julius Fischer^1^, Julius Steffen^1,2^, Sven Peterß^3^, Stefan Kääb^1,2^, Moritz Sinner^1,2^, Korbinian Lackermair^1^, Martin Orban^1,2^, Steffen Massberg^1,2^ and Clemens Scherer^1,2 *^

(#) = contributed equally

**Affiliations**

(1) Department of Medicine I, LMU University Hospital, LMU Munich, Germany

(2) DZHK (German Centre for Cardiovascular Research), Munich Heart Alliance, Munich, Germany

(3) Department of Cardiac Surgery, LMU University Hospital, LMU Munich, Germany

**Address of Correspondence**

PD Dr. med. Clemens Scherer

Department of Medicine I,

University Hospital, LMU Munich

Ludwig-Maximilians-University Munich

Marchioninistraße 15, 81377 Munich, Germany

E-Mail: clemens.scherer@med.uni-muenchen.de

**Supplemental tables**

**Table S1 – Further LMUshock registry patient characteristics**

|  | Non-TTP  (n = 1377) | TTP  (n = 184) | p-value |
| --- | --- | --- | --- |
| **Vital parameters at admission, median [IQR]** |  |  |  |
| Heart rate [bpm] | 100.00 [84.00, 116.00] | 96.00 [80.00, 116.00] | 0.15 |
| Systolic blood pressure [mmHg] | 85.00 [75.00, 97.00] | 87.00 [75.00, 100.00] | 0.42 |
| Diastolic blood pressure [mmHg] | 49.00 [40.00, 55.00] | 45.00 [38.00, 54.00] | 0.14 |
| **Cardiac arrest, n (%)** | 765 (55.6) | 128 (69.6) | <0.01* |
| Extra-hospital cardiac arrest | 380 (27.6) | 47 (25.5) | 0.06 |
| **Laboratory parameters at admission, median [IQR]** |  |  |  |
| Arterial pH | 7.30 [7.20, 7.38] | 7.29 [7.19, 7.38] | 0.80 |
| Lactate [mmol/l] | 4.50 [2.20, 10.30] | 5.14 [2.30, 10.04] | 0.65 |
| Hemoglobin [mg/dl] | 10.90 [10.10, 12.70] | 10.55 [10.00, 12.12] | 0.02* |
| Troponin T (hs) [ng/ml] | 36.30 [3.43, 175.00] | 18.25 [3.13, 151.00] | 0.39 |
| Creatine Kinase [U/l] | 507.00 [145.50, 1852.75] | 751.00 [201.00, 2377.00] | 0.20 |
| Creatinine, Jaffé [mg/dl] | 1.40 [1.10, 2.00] | 1.40 [1.10, 1.98] | 0.56 |
| **Intensive care treatment, n (%)** |  |  |  |
| Catecholamines/Vasopressors | 1292 (93.8) | 179 (97.3) | 0.09 |
| Veno-arterial ECLS | 372 (27.0) | 57 (31.0) | 0.30 |
| Ventricular coaxial pump | 144 (10.5) | 24 (13.0) | 0.35 |
| Dialysis | 353 (25.6) | 56 (30.4) | 0.19 |
| **Events within ICU treatment, n (%)** |  |  |  |
| Bleeding > BARC 2 | 201 (14.6) | 39 (21.2) | 0.03* |
| Intracerebral bleeding | 91 (6.6) | 20 (10.9) | 0.05 |
| Ischemic stroke | 145 (10.5) | 20 (10.9) | 0.99 |
| Hemorrhagic stroke | 14 (1.0) | 2 (1.1) | 1.00 |
| Sepsis | 213 (15.5) | 30 (16.3) | 0.85 |

All values are presented as mean/median and confidence intervals, or absolute values and percentages.

ECLS = extracorporeal life support, pH = potential of hydrogen, BARC = Bleeding Academic Research Consortium.

* = p-values <0.05 were considered statistically significant

**Table S2 – Further characteristics in AMI-CS patients**

|  | AMI-CS-non-TTP  (n = 689) | AMI-CS-TTP  (n = 83) | p-value |
| --- | --- | --- | --- |
| **Vital parameters at admission, median [IQR]** |  |  |  |
| Heart rate [bpm] | 100.00 [85.00, 120.00] | 96.00 [82.00, 116.00] | 0.12 |
| Systolic blood pressure [mmHg] | 85.00 [70.00, 97.00] | 90.00 [75.00, 100.00] | 0.65 |
| Diastolic blood pressure [mmHg] | 47.00 [40.00, 55.00] | 48.00 [39.00, 55.00] | 0.88 |
| **Cardiac arrest, n (%)** | 463 (67.2) | 66 (79.5) | 0.03* |
| Extra-hospital cardiac arrest | 240 (34.8) | 25 (30.1) | 0.83 |
| **Catecholamines/Vasopressors, n (%)** | 654 (94.9) | 81 (97.6) | 0.42 |
| **Veno-arterial ECLS, n (%)** | 221 (32.1) | 31 (37.3) | 0.40 |
| **Ventricular coaxial pump, n (%)** | 86 (12.5) | 16 (9.3) | 0.12 |
| **Dialysis within ICU treatment, n (%)** | 170 (24.7) | 22 (26.5) | 0.82 |
| **Events within ICU treatment, n (%)** |  |  |  |
| Bleeding > BARC 2 | 117 (17.0) | 17 (20.5) | 0.52 |
| Intracerebral bleeding | 52 (7.5) | 12 (14.5) | 0.05 |
| Ischemic stroke | 60 (8.7) | 13 (15.7) | 0.06 |
| Hemorrhagic stroke | 6 (0.9) | 1 (1.2) | 1.00 |
| Sepsis | 90 (13.1) | 9 (10.8) | 0.69 |

All values are presented as mean/median and confidence intervals, or absolute values and percentages.

ECLS = extracorporeal life support, BARC = Bleeding Academic Research Consortium.

* = p-values <0.05 were considered statistically significant

**Table S3 – Further characteristics in non-AMI-CS patients**

|  | non-AMI-CS-non-TTP  (n = 714) | non-AMI-CS-TTP  (n = 75) | p-value |
| --- | --- | --- | --- |
| **Vital parameters at admission, median [IQR]** |  |  |  |
| Heart rate [bpm] | 96.00 [81.00, 114.00] | 93.00 [80.00, 114.25] | 0.33 |
| Systolic blood pressure [mmHg] | 88.00 [75.00, 97.00] | 90.00 [80.00, 100.00] | 0.20 |
| Diastolic blood pressure [mmHg] | 50.00 [40.00, 55.00] | 45.50 [35.25, 51.00] | 0.09 |
| **Cardiac arrest, n (%)** | 322 (45.1) | 42 (56.0) | 0.19 |
| Extra-hospital cardiac arrest | 145 (20.3) | 17 (22.7) | <0.01* |
| **Catecholamines/Vasopressors, n (%)** | 664 (93.0) | 72 (96.0) | 0.46 |
| **Veno-arterial ECLS, n (%)** | 161 (22.5) | 16 (21.3) | 0.92 |
| **Ventricular coaxial pump, n (%)** | 61 (8.5) | 5 (6.7) | 0.73 |
| **Dialysis within ICU treatment, n (%)** | 191 (26.8) | 26 (34.7) | 0.19 |
| **Events within ICU treatment, n (%)** |  |  |  |
| Bleeding > BARC 2 | 88 (12.3) | 18 (24.0) | 0.01* |
| Intracerebral bleeding | 40 (5.6) | 7 (9.3) | 0.30 |
| Ischemic stroke | 86 (12.0) | 6 (8.0) | 0.40 |
| Hemorrhagic stroke | 9 (1.3) | 0 (0.0) | 0.68 |
| Sepsis | 128 (17.9) | 16 (21.3) | 0.57 |

All values are presented as mean/median and confidence intervals, or absolute values and percentages.

ECLS = extracorporeal life support, BARC = Bleeding Academic Research Consortium.

* = p-values <0.05 were considered statistically significant

**Table S4 – Uni- and multivariable regression analysis for AMI-CS patients**

|  | **Univariable**  OR | 95% CI | p | **Multivariable**  OR | 95% CI | p-value |
| --- | --- | --- | --- | --- | --- | --- |
| **Age, years** | 1.01 | 0.30 – 3.35 | 0.59 |  |  |  |
| **Sex, male** | 1.01 | 0.59 – 1.72 | 0.98 |  |  |  |
| **Creatine at admission, mg/dl** | 1.07 | 0.89 – 1.31 | 0.44 |  |  |  |
| **Lactate at admission mmol/l** | 1.03 | 1.00 – 1.07 | 0.08 | 1.03 | 1.00 – 1.07 | 0.07 |
| **Creatine kinase at admission, U/l** | 1.00 | 1.00 – 1.00 | 0.67 |  |  |  |
| **pH at admission** | 0.59 | 0.12 – 2.91 | 0.52 |  |  |  |
| **Culprit lesion RCA** | 2.47 | 1.50 – 4.02 | <0.001* | 2.52 | 1.54 – 4.11 | <0.001* |

Univariable and multivariable regression analysis regarding TTP therapy during ICU stay.

pH = potential of hydrogen, RCA = right coronary artery.

* = p-values <0.05 were considered statistically significant.

**Table S5 - Uni- and multivariable regression analysis for non-AMI-CS patients**

|  | **Univariable**  OR | 95% CI | p | **Multivariable**  OR | 95% CI | p-value |
| --- | --- | --- | --- | --- | --- | --- |
| **Age, years** | 1.02 | 1.01 – 1.05 | <0.001* | 1.03 | 1.01 – 1.05 | <0.005* |
| **Sex, male** | 0.60 | 0.37 – 0.99 | 0.04* | 0.67 | 0.40 – 1.13 | 0.13 |
| **Creatine at admission, mg/dl** | 1.06 | 0.91 – 1.25 | 0.41 | 1.15 | 0.97 – 1.36 | 0.10 |
| **Lactate at admission mmol/l** | 0.98 | 0.93 – 1.03 | 0.42 |  |  |  |
| **Creatine kinase at admission, U/l** | 1.00 | 1.00 – 1.00 | 0.95 |  |  |  |
| **pH at admission** | 0.99 | 0.18 – 5.35 | 0.99 |  |  |  |
| **Etiology of cardiogenic shock** |  |  |  |  |  |  |
| Cardiomyopathy | 0.23 | 0.09 – 0.50 | <0.001* | 0.26 | 0.11 – 0.63 | <0.005* |
| Intoxication | 1.88 | 0.36 – 9.56 | 0.45 | 4.45 | 0.75 – 26.30 | 0.10 |
| Myocarditis | 2.10 | 0.84 – 5.27 | 0.11 | 3.21 | 1.19 – 8.64 | 0.02* |
| Valvular | 1.12 | 0.58 – 2.15 | 0.73 | 0.85 | 0.43 – 1.66 | 0.63 |
| Other | 0.50 | 0.24 – 1.03 | 0.06 | 0.45 | 0.22 – 0.95 | 0.04* |
| Unknown | 1.88 | 0.37 – 9.65 | 0.75 | 1.60 | 0.30 – 8.49 | 0.58 |

Univariable and multivariable regression analysis regarding TTP therapy during ICU stay.

pH = potential of hydrogen.

* = p-values <0.05 were considered statistically significant.

**Supplemental Figures**

**Figure S1: 30-day survival in TTP patients with and without AMI-CS**

Cumulative survival curves for patients with TTP therapy because of bradycardia secondary to acute myocardial infarction complicated by cardiogenic shock (AMI-CS-TTP) (red) and CS patients with TTP therapy due to all other causes (non-AMI-CS-TTP) (black) for 30 days from ICU admission. p = 0.0032


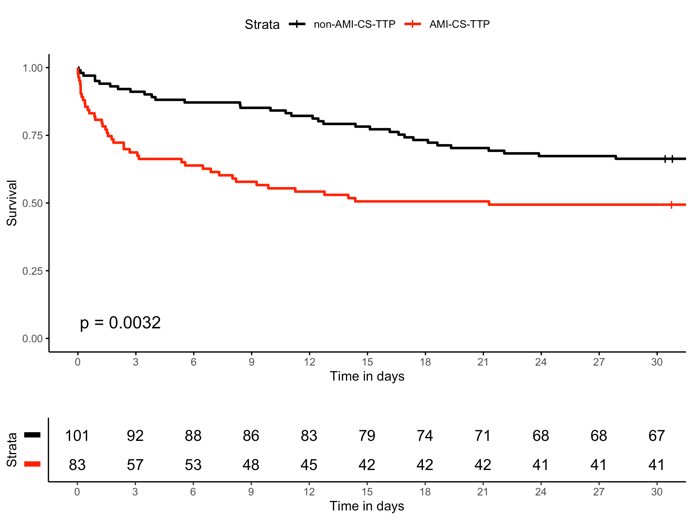


**Figure S2: 30-day survival in non-AMI-CS patients with and without TTP treatment**

Cumulative survival curves for patients without acute myocardial infarction complicated by cardiogenic shock with (red) and without (black) TTP treatment for 30 days from ICU admission. p = 0.62


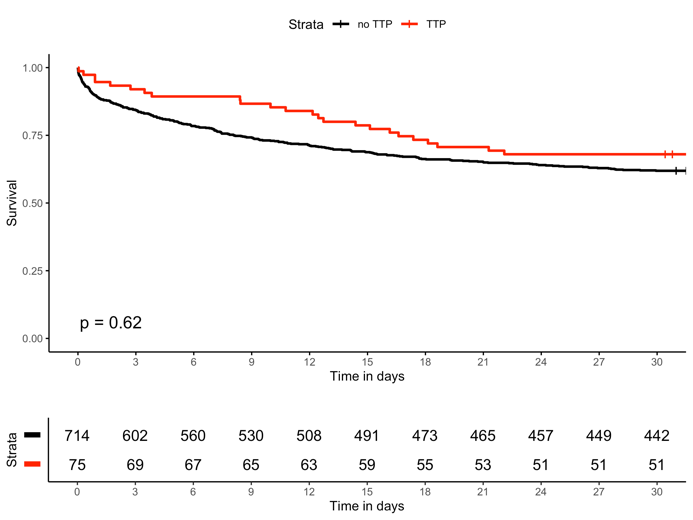

Supplement: Supplementary file 1 — Supplementary Information. [file 41598_2025_10364_MOESM1_ESM.docx]
